# Prevalence and risk factors of asymptomatic colorectal diverticulosis in Taiwan

**DOI:** 10.1186/s12876-015-0267-5

**Published:** 2015-04-01

**Authors:** Fu-Wei Wang, Hung-Yi Chuang, Ming-Shium Tu, Tai-Ming King, Jui-Ho Wang, Chao-Wen Hsu, Ping-I Hsu, Wen-Chi Chen

**Affiliations:** 1 Department of Family Medicine, Kaohsiung Veterans General Hospital, Kaohsiung City, 81362 Taiwan; 2 Department of Public Health, Kaohsiung Medical University, Kaohsiung City, 803 Taiwan; 3 Division of Colorectal Surgery, Department of Surgery, Kaohsiung Veterans General Hospital, Kaohsiung City, 81362 Taiwan; 4 Division of Gastroenterology, Department of Internal Medicine, Kaohsiung Veterans General Hospital, 386 Ta-Chung 1 st Road, Kaohsiung City, 80424 Taiwan; 5 School of Medicine, National Yang-Ming University, Taipei, Taiwan

**Keywords:** Colorectal diverticulosis, Asymptomatic, Colonoscopy, Health check-up

## Abstract

**Background:**

To investigate the prevalence and risk factors of asymptomatic colorectal diverticulosis in Taiwanese general population.

**Methods:**

From January 2009 to December 2011, consecutive asymptomatic subjects undergoing a health check-up were evaluated by colonoscopy. The colorectal diverticulosis was assessed, and a medical history and demographic data were obtained from each subject. Logistic regression analysis was conducted to search the risk factors of colorectal diverticulosis.

**Results:**

Of the 1899 asymptomatic subjects, the prevalence of colorectal diverticulosis was 13.5%. On univariate logistic regression analysis, age over 60 years old, male, adenomatous polyp, current smoking and heavy alcohol consumption were significantly associated with diverticulosis. Multivariate logistic regression analysis revealed that age over 60 years old (relative risk [RR], 2.57; 95% confidence interval [CI], 1.64-6.47), adenomatous polyps (RR, 2.18; 95% CI, 1.18-4.61) and heavy alcohol consumption (RR, 1.82; 95% CI, 1.04-3.08) were independent predictors for colorectal diverticulosis.

**Conclusions:**

The prevalence of asymptomatic colorectal diverticulosis was 13.5% in Taiwan. Age over 60 years old, adenomatous polyp and heavy alcohol consumption may affect the risk of development of the disease.

## Background

A diverticulum is a herniation through a weak site of the bowel wall that produces a small outpouching [1]. Although largely asymptomatic, this condition constitutes a considerable public health burden. It is estimated that 10% to 30% of patients with diverticulosis will suffer from complications such as diverticulitis and gastrointestinal bleeding and the associated mortality is estimated at 23,600 death per year in Europe [2,3]. Recently, diverticular disease is also found to be associated with an increased risk of subsequent arterial and venous thromboembolic events [4]. Identification of the risk factors for colorectal diverticulosis could provide both risk stratification and development of risk reduction strategies.

Colorectal diverticulosis shows both geographic and ethnic variability. It is rare in Africa and Asia but common in the United States, Europe and Australia [5,6]. In Western countries, colonic diverticula occur mainly in the sigmoid [7,8], whereas in Asian patients, the right side of the colon is more commonly involved [9,10]. Several risk factors for colorectal diverticulosis in the Western countries have been indentified previously such as old age, moderate to heavy alcohol use, constipation and lower fiber diet [11-13]. However, only a few studies have been conducted to investigate the risk factors for colorectal diverticulosis in Asian countries. Recently, a cross-sectional study from Japan showed an association between colorectal diverticulosis and old age, heavy alcohol consumption and smoking, and atherosclerosis disease [14] but a prospective study in Korea showed no association between colorectal diverticulosis and body mass index (BMI), lower fiber diet and a history of cigarette smoking [15].

Currently, the risk factors for colorectal diverticulosis remain unclear and the results of risk factors for colorectal diverticulosis in Asian population are quite conflicting. Some people in Taiwan underwent health check-up including colonoscopy at their own expense because of various reasons. We therefore conducted this cross-sectional study to investigate the risk factors for colorectal diverticulosis in Taiwan.

## Methods

### Subjects

From January 2009 to December 2011, consecutive asymptomatic subjects aged ≥ 20 years old undergoing a colonoscopy during a health check-up were included into this study. The subjects were excluded if they reported symptoms of lower gastrointestinal tract disease including rectal bleeding, a marked change in bowel habits, or lower abdominal pain that would normally require medical evaluation. Other exclusion criteria were a history of colitis, colorectal polyps or colorectal cancer, prior colonic surgery, undergoing a sigmoidscopy, a colonoscopy, or a barium enema within the previous 10 years. The study protocol was approved by Institutional Review Board at Kaohsiung Veterans General Hospital and all participants provided written informed consent.

### Study design

A complete history and physical examination were performed for each subject undergoing the health check-up. All subjects were carefully queried regarding the presence of abdominal symptoms in the previous 1 month. Subjects who responded negatively were classified as asymptomatic subjects and were enrolled into this study. All the participants received anthropometric and blood biochemical tests including fasting plasma glucose, serum triglyceride and high-density lipoprotein (HDL)–cholesterol level, and underwent total colonoscopy. Colonoscopies were performed by three experienced endoscopists (King TM, Wang JH, and Hsu CW) using the Olympus PCF-Q240AL and PCF-Q260AL endoscopy (Olympus Corp., Tokyo, Japan) after the subjects had fasted overnight. Bowel preparation was performed with oral saline lexative following the protocol of diagnostic colonoscopy. The patients were carefully examined for colorectal mucosal lesion. If colorectal diverticula were observed, their location and type was recorded carefully. Distribution type was defined as the following: right-side colon, involving the cecum, ascending colon, or transverse colon; left- side colon, involving the splenic flexure, descending colon, sigmoid colon, or rectum; or bilateral, involving the entire colon. Colorectal polyp was defined as a protuberance into the lumen from the normally flat colonic mucosa. All visible polyps were removed and examined histologically by the pathologist. The pathology types of colorectal polyps were subsequently categorized into hyperplastic polyps and adenomatous polyps.

To assess the relationship between clinical characteristics and asymptomatic colorectal diverticulosis, the following data were recorded for each subject: age; gender; educational status; consumption of tobacco, alcohol, coffee, tea, spicy foods or betel nut, exercise habit, whether vegetarian or not and long-term use of non-steroidal anti-inflammatory drug (NSAID). All variables were categorized for data analyses.

### Statistical analysis

The chi-square test or Fisher’s exact test was employed to investigate the relationship between the rate of colorectal diverticulosis and clinical characteristics. These variables included the following: gender; age (<39, 40–49, 50–59, 60–69 or >70 years); education status (<10, 10–12, or >12 years); BMI (<25, 25–30, or >30); regular NSAID use at least 1 year (yes or no); colorectal polyps (hyperplastic polyps or adenomatous polyps); smoking status (no, former smoking, current smoking); consumption of alcohol ,coffee, tea or spicy foods and exercise habit (no, ≤ 3 times per week, or >3 times per week); betel nut habit and vegetarian (yes or no). Metabolic syndrome was defined according to the modified National Cholesterol Education Program Adult Treatment Panel III definition for South Asians and Chinese. A *p* value less than 0.05 was considered significant. Significant variables revealed by univariate analysis were subsequently assessed by a stepwise logistic regression method to identify independent clinical factors predicting the presence of colorectal diverticulosis. All statistical analyses were performed using SPSS version 17.0 (SPSS Inc. Chicago, II, USA ).

## Results

### Patient demographics and colonoscopic characteristics

**Figure 1:**
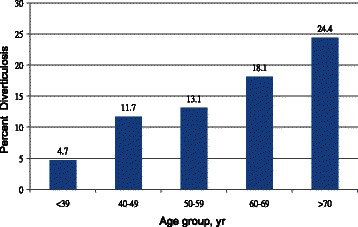
**Prevalence of asymptomatic diverticulosis stratified by age group.**

From January 2009 to December 2011, 1899 asymptomatic subjects (mean age, 52.8 ± 10.6 years; age range, 20–86 years; male/female, 1203/696) was recruited into this study. Among them, 256 subjects (13.5%) had colorectal diverticulosis. Diverticula were located predominately in the right-side of the colon in 52.3% (n = 134), left-side in 25% (n = 64), and bilaterally in 22.7% (n = 58) of the subjects (Table 1). The prevalence of colorectal diverticulosis increased with age (Figure 1). The prevalence of hyperplastic polyps and adenomatous polyps were 11.1%, 16.1%, respectively.

**Table 1:** **Demographics and endoscopic findings of asymptomatic health check-up subjects (N = 1,899)**

Clinical characteristics	
Age, n (%)	
Mean (SD) (years)	52.8 (10.6)
< 39	193 (10.2)
40-49	557 (29.3)
50-59	681 (35.9)
60-69	337 (17.8)
> 70	131 (6.8)
Height, cm	165.8 (8.2)
Weight, kg	66.2 (12.3)
Gender, n (%)	
Men	1203 (63.2)
Women	696 (36.8)
Body mass index, n (%)	
Mean (SD)	23.9 (3.4)
< 25	1233 (64.9)
25-30	573 (30.2)
> 30	93 (4.9)
Education level, n (%)	
Middle school	144 (7.6)
High school	799 (42.1)
University	641 (33.7)
Graduate school	315 (16.6)
Yearly income (US dollars), n (%)	
< 10,000	182 (9.6)
10,000 - 30,000	984 (51.8)
> 30,000	733 (38.6)
Colonscopic findings, n (%)	
Hyperplastic polyp	210 (11.1)
Adenomatous polyp	305 (16.1)
Diverticulosis	256 (13.5)
Location	
Right side only	134 (52.3)
Left side only	64 (25.0)
Both sides	58 (22.7)

### Risk factors for the development of colorectal diverticulosis

Table 2 showed the results of univariate analysis for the risk factors of colorectal diverticulosis. Current smoking, old age, male gender, adenomatous polyps and heavy alcohol consumption were significantly associated with the presence of colorectal diverticulosis (p = 0.045, 0.005, 0.001, 0.002, <0.001 respectively). The subjects with and without colorectal diverticulosis had comparable education level, BMI, coffee, tea, spicy food consumption, betel nut chewing, exercise habit, vegetarian, metabolic syndrome status, and NSAID use. Multivariate analysis with stepwise logistic regression showed that age greater than 60 years (RR, 2.57, 95% CI, 1.64-6.47), heavy alcohol consumption (RR, 1.82, 95% CI, 1.04-3.08) and presence of adenomatous polyps (RR, 2.18, 95% CI, 1.18-4.61) were independent predictors for asymptomatic colorectal diverticulosis (Table 3).

**Table 2:** **Univariate analysis of the risk factors for the development of colorectal diverticulosi**s

Principal parameter	Diverticulosis (+)	Diverticulosis (−)	*P*value
Sex, n (%)			0.001
Men	193 (75.4)	1010 (61.5)	
Women	63 (24.6)	633 (38.5)	
Age (yr), n (%)			0.005
< 39	9 (3.5)	184 (11.2)	
40-49	65 (25.4)	492 (29.9)	
50-59	89 (34.8)	592 (36.0)	
60-69	61 (23.8)	276 (16.8)	
> 70	32 (12.5)	99 (6.1)	
Education (yr), n (%)			0.136
< 10	56 (21.8)	338 (20.6)	
10-12	103 (40.1)	723 (44.0)	
> 12	97 (38.1)	582 (35.4)	
BMI,* n (%)			0.248
< 25	161 (62.9)	1072 (65.2)	
25-30	80 (31.2)	493 (30.1)	
> 30	15 (5.9)	78 (4.7)	
NSAID^†^ use, n (%)			0.636
No	247 (96.5)	1572 (95.7)	
Yes	9 (3.5)	71 (4.3)	
Colon polyps			0.002
None	146 (57.1)	1233 (75.3)	
Hyperplastic polyp	38 (14.8)	172 (10.5)	
Adenomatous polyp	72 (28.1)	233 (14.2)	
Smoking status, n (%)			0.045
Never smoking	144 (56.3)	1068 (65.0)	
Former smoking	33 (12.9)	238 (14.5)	
Current smoking	79 (30.8)	337 (20.5)	
Alcohol drinking, n (%)			<0.001
No	155 (60.6)	1231 (74.9)	
≦ 3 times per week	55 (21.6)	320 (19.5)	
> 3 times per week	46 (17.8)	92 (5.6)	
Coffee drinking, n (%)			0.724
No	144 (56.3)	907 (55.2)	
≦ 3 times per week	46 (18.0)	311 (18.9)	
> 3 times per week	66 (25.7)	425 (25.9)	
Tea drinking, n (%)			0.486
No	98 (38.2)	677 (41.2)	
≦ 3 times per week	53 (20.6)	311 (18.9)	
> 3 times per week	105 (41.2)	655 (39.9)	
Spicy foods consumption, n (%)			0.269
No	137 (53.4)	876 (53.3)	
≦ 3 times per week	74 (28.9)	434 (26.4)	
> 3 times per week	45 (17.7)	333 (20.3)	
Betel nut use, n (%)			0.428
No	238 (92.8)	1556 (94.7)	
Yes	18 (7.2)	87 (5.3)	
Exercise habit, n (%)			0.667
No	95 (37.0)	550 (33.5)	
≦ 3 times per week	72 (28.2)	490 (29.8)	
> 3 times per week	89 (34.8)	603 (36.7)	
Vegetarian, n (%)			0.375
No	248 (96.9)	1579 (96.1)	
Yes	8 (3.1)	64 (3.9)	
Metabolic syndrome, n (%)			0.369
No	164 (63.9)	1099 (66.9)	
Yes	92 (36.1)	544 (33.1)	

*BMI = body mass index, indicating weight in kg divided by body surface area. ^†^NSAID, non-steroid anti-inflammatory drug.

**Table 3:** **Multi-variate analysis of risk factors for colorectal diverticulosis**

Variable	Relative risk	95% CI^†^	*P*value
Age >60 y/o	2.57	1.64-6.47	0.001
Alcohol drinking >3 times per week	1.82	1.04-3.08	0.023
Colorectal Adenomatous polyps	2.18	1.18-4.61	0.005

^†^CI := confidence interval.

## Discussion

The current study demonstrated that the prevalence of colorectal diverticulosis were 13.5% in asymptomatic Taiwanese. Old age, heavy alcohol consumption and adenomatous polyps are risk factors of colorectal diverticulosis. To our knowledge, this is the first work investigating the prevalence and risk factors of colorectal diverticulosis in an asymptomatic Taiwanese population based on colonoscopic findings.

The location of colorectal diverticulosis in subjects from this study was also different from that of Western countries [16]. In Western countries, diverticula occur mainly at left side of the colon and only 15% occur in the right side [10]. In this study, diverticula were primarily located in the right side of the colon (52.3%) and only 25% were in the left side. In other Asian countries, diverticula are also predominantly located in the right side of the colon [17]. Why diverticulosis is predominantly right-sided in Asian people compared with other populations is unclear. It is possible that the sensitivity of the colon to environmental factors varies with the characteristics such as the length and muscle thickness of the colon, body weight, and the structure of the neural and humoral systems [6]. The incidence of colorectal diverticulosis appears to increase with increasing age [11]. In Western countries, the prevalence of colorectal diverticulosis was less than 10% in individuals younger than 40 years old and greater than 50% in individuals older than 70 years old [16]. In our study, the prevalence of colorectal diverticulosis also increased with age (4.7% in individuals younger than 40 years old vs. 24.4% in individuals older than 70 years old). It could be explained by the fact that the tensile strength of the colon wall declines with the increase of age [1]. In addition, abnormal thickness of muscles of colonic wall, including collagen cross-linking, is promoted by abnormal colonic movement due to a lack of dietary fiber and results in increase of intraluminal colonic pressure of the thickened muscles which changes with the increase of age [17].

This study revealed a strong positive association between colorectal adenomatous polyps and diverticulosis by univariate and multivariate analysis. The association of colorectal diverticulosis and the risk of colorectal adenomas is controversial. Morini et al. found significantly more adenoma/advanced adenoma in the sigmoid colon of patients with diverticula than in controls in a study of Italian patients undergoing colonoscopy [18]. In a retrospective study of Japanese patients undergoing total colonoscopy, Hierta et al. found that on multivariate analysis adjusted for age and sex there was a significant association between colorectal diverticulosis and colon polyps in all locations [19]. In contrast, Meurs-Szojda et al. found in 4,241 patients that there was no relation between patients with diverticulosis and a higher incidence of colorectal polyps when using an age-stratified analysis [20]. Colorectal adenomatous polyps are apparently not a causative factor of colorectal diverticulosis but may be associated with diverticulosis by sharing one or more common risk factors such as age, high-fiber diet, and alcohol.

Our study also showed that consumption of alcoholic drinks three times or more per week was an independent predictor for colorectal diverticulosis in asymptomatic subjects. A Danish study found a relative risk of 2 for alcoholics to have admissions due to colonic diverticular disease [21]. Song et al. revealed that alcohol drinkers were two times (RR:2.2) more likely to develop diverticulosis than nondrinkers when assessed by multivariate analysis in a colonoscopy-based study [15]. The exact mechanism whereby alcohol consumption could increase the risk of colorectal diverticulosis is unknown. However, an effect on colorectal motility by alcohol [22], a direct damaging effect via oxidative stress on colonic deoxyribonucleic acid (DNA) [23] or one mediated via the colonic flora’s alcohol dehydrogenase (ADH) may play a role [24]. Berenson et al. reported that intravenous administration of alcohol consistently decreases rectosigmoid motor activity, which correlates inversely with serum alcohol levels in humans [25]. Furthermore, alcohol withdrawal in chronic alcohol users leads to significant improvement in colorectal transit time as a result of an exclusive increase in rectosigmoidal transit time [22].

Despite of the findings, this study had certain limitations. First, self-selection bias of the population in this trial was possible because all enrolled subjects underwent self-paid health examination, which usually indicated better economic status than the general population in Taiwan (Table 1). Second, the studied subjects may differ from the subjects in primary care hospital because our hospital is a tertiary care center. Third, the limited information on type and duration of alcohol use and the lack of a detailed dietary history with regard to fiber and fat intake might decrease the power of these factors.

## Conclusion

The prevalence of colorectal diverticulosis is 13.5% in this cross-section study of asymptomatic Taiwanese subjects, with 52.3% of the cases on the right side of the colon. Advanced age and alcohol consumption are identifiable risk factors for the development of colorectal diverticulosis. These findings suggested that lifestyle modification including abstaining from alcohol might potentially decrease the risk of developing colorectal diverticulosis. Colorectal adenomatous polyps was also associated with the presence of colorectal diverticular disease but the causative relationship is vague. Further studies are warranted to investigate the pathophysiological mechanisms underlying the association of alcohol consumption with the risk of colorectal diverticulosis.

## Abbreviations

RR: Relative risk
CI: Confidence interval
BMI: Body mass index
HDL: High-density lipoprotein
NSAID: Non-steroidal anti-inflammatory drug
DNA: Deoxyribonucleic acid
ADH: Alcohol dehydrogenase
